# Cortical thickness, gyrification and sulcal depth in trigeminal neuralgia

**DOI:** 10.1038/s41598-021-95811-z

**Published:** 2021-08-11

**Authors:** Meng Li, Jianhao Yan, Hua Wen, Jinzhi Lin, Lianbao Liang, Shumei Li, Shuangcong Xie, Wuming Li, Chulan Lin, Guihua Jiang

**Affiliations:** 1 The Department of Medical Imaging, Guangdong Second Provincial General Hospital, Guangzhou, Guangdong , People’s Republic of China; 2 The Department of Neurosurgery, Guangdong Second Provincial General Hospital, Guangzhou, Guangdong People’s Republic of China

**Keywords:** Neuroscience, Neurology

## Abstract

Neuroimaging studies have documented brain structural alterations induced by chronic pain, particularly in gray matter volume. However, the effects of trigeminal neuralgia (TN), a severe paroxysmal pain disorder, on cortical morphology are not yet known. In this study, we recruited 30 TN patients and 30 age-, and gender-matched healthy controls (HCs). Using Computational Anatomy Toolbox (CAT12), we calculated and compared group differences in cortical thickness, gyrification, and sulcal depth with two-sample t tests (p < 0.05, multiple comparison corrected). Relationships between altered cortical characteristics and pain intensity were investigated with correlation analysis. Compared to HCs, TN patients exhibited significantly decreased cortical thickness in the left inferior frontal, and left medial orbitofrontal cortex; decreased gyrification in the left superior frontal cortex; and decreased sulcal depth in the bilateral superior frontal (extending to anterior cingulate) cortex. In addition, we found significantly negative correlations between the mean cortical thickness in left medial orbitofrontal cortex and pain intensity, and between the mean gyrification in left superior frontal cortex and pain intensity. Chronic pain may be associated with abnormal cortical thickness, gyrification and sulcal depth in trigeminal neuralgia. These morphological changes might contribute to understand the underlying neurobiological mechanism of trigeminal neuralgia.

## Introduction

Trigeminal neuralgia (TN) is a common paroxysmal facial pain disorder with annual incidence of approximately 4.3–27 per 100,000 people^1–3^. TN is mainly manifested as recurrent electric shock-like attacks in the distribution of one or more trigeminal nerve branches^4^. These attacks can be commonly evoked by subtle, harmless stimuli or occur spontaneously.

Although neurobiological mechanisms of TN are not fully understood, neuroimaging studies have supported that chronic pain is associated with brain morphological changes, particularly in gray matter volume^5–9^. For example, based on the voxel-based morphometry (VBM), Obermann et al.^7^ found decreased gray matter volume in TN patients, which were mainly located in the dorsolateral prefrontal cortex, anterior cingulate cortex (ACC), primary/secondary somatosensory and orbitofrontal cortices, thalamus, insula, and cerebellum compared with controls. Wang et al.^8^ reported that patients with classic TN displayed gray matter volume reductions and negatively correlations between gray matter volume of left inferior temporal gyrus and pain intensity or disease duration in patients. Tsai et al.^6^ investigated the left and right TN patients respectively, and found gray matter volume reductions in the inferior frontal gyrus, precentral gyrus, cerebellum, thalamus, ventral striatum, and putamen among left TN patients and in the prefrontal cortex, precentral gyrus, cerebellar tonsil, thalamus, hypothalamus, and nucleus accumbens among right TN patient. However, the effects of TN on cortical morphology remain enigmatic.

Cortical characteristics, including cortical thickness, gyrification index and sulcal depth in the present study, have been indicated to reflect the regulation of intermediate progenitor cells genesis and amplification^10^, and cortical morphological analyses are widely used to investigate the neuroplastic changes associated with aging^11,12^, cognitive performance^13^, and pathophysiological changes^14,15^. Based on the surface-based morphometry (SBM), previous studies have investigated alterations of cortical thickness in TN^16–18^. For example, DaSilva et al.^16^ chose cortical regions based on sensory, motor, and emotional processing of pain as ROIs, and found abnormal cortical thickness in sensorimotor regions and emotional regions. Parise et al.^17^ used SBM and reported cortical thickness reductions in the left cuneus and left fusiform in the TN patients. In contrast, the study of DeSouza et al.^18^ showed that the TN patients had cortical thickening in the contralateral primary somatosensory cortex and frontal pole, while thinning in the pregenual anterior cingulate cortex, the insula and the orbitofrontal cortex compared to controls. Although previous studies have found cortical abnormalities in TN patients, these studies have not produced consistent results and these differences may be the result of differences in the precise patient samples studied or methods used. Furthermore, gyrification and sulcal depth, another two morphological characteristics, that are thought to be parallel to cortical thickness^19,20^, have fewer been investigated in TN patients, except one study of Wang et al.^21^, which reported significant reductions of local gyrification index in the left insular cortex. Sulcal depth is sensitive to cortical atrophy and development^22–24^, and the spatial distribution of deep sulcal regions is relatively robust^25,26^. Cortical gyrification adapts the cortical surface area to the skull, which promotes the development of neural circuits^27^. Thus, the exploration of cortical morphology might provide more accurate and reliable information about underling neurophysiological mechanisms related to chronic pain.

Therefore, the aim of present study was to perform a comprehensive analysis on alterations of cortical morphology in TN patients and explore the relationships between cortical characteristic’s changes and pain duration or intensity in the TN patients. Given the findings of previous neuroimaging studies of chronic pain, we hypothesized that cortical characteristics would be altered in some brain regions in the TN patients^5–7^. In addition, we also hypothesized that the cortical characteristics in significant regions observed in TN may be correlated with the pain duration or intensity.

## Materials and methods

### Participants

Thirty TN patients (mean age: 51.63 ± 8.16 years, 18 females, right-handed) were recruited from the Guangdong Second Provincial General Hospital. All TN patients were screened according to the International Classification of Headache Disorders version III criteria^4^ to diagnose of TN and assess the intensity and frequency of the symptoms using visual-analog scales (VAS). Most of the patients were treated with carbamazepine and five were treated with gabapentin due to allergies (Table 1). At time of study inclusion, all patients had active unilateral pain in one or more branches (the ophthalmic [V1], the maxillary [V2], and the mandibular [V3]) of the trigeminal nerve. Exclusion criteria were: (1) chronic pain other than TN (e.g., tension-type headache or migraine); (2) patients with neural-associated diseases; (3) TN patients with brain surgical treatment. Thirty age- and gender-matched healthy controls (HC, mean age: 49.80 ± 9.31 years, 13 females, right-handed) were also recruited for this study.

**Table 1:** Demographic and clinical characteristics of the trigeminal neuralgia (TN) patients and healthy controls (HCs).

Characteristics	TN (n = 30)	HC (n = 30)	*p*-value
Age (years)	51.63 ± 8.16	49.80 ± 9.31	0.42
Gender (female/male)	18/12	13/17	0.20
Education (years)	9.50 ± 3.10	9.93 ± 3.67	0.62
Total intracranial volume (cm^3^)	1432.2 ± 109.1	1471.9 ± 135.5	0.18
Pain intensity	8.83 ± 1.23	N/A	
Affected side	8 left / 22 right	N/A	
Pain Dist (V1/V2/V3%)	16/73/67	N/A	
TN duration (years)	4.72 ± 3.69	N/A	
Age of Pain Onset	46.82 ± 9.72	N/A	
Medication	25 Carbamazepine/5 Gabapentin	N/A	

This study was approved by the Ethics Committee of Guangdong Second Provincial General Hospital. Written informed consent was obtained from each subject. The study was carried out in accordance with the relevant guidelines and regulations.

### Data acquisition

Imaging data were obtained on a 3.0 T Philips Ingenia MR scanner using a 32-channel head coil at the department of Medical Imaging in Guangdong Second Provincial General Hospital. High resolution T1-weighted 3D images were collected using a fast field echo (FFE) pulse sequence with repetition time (TR) = 7.9 ms, echo time (TE) = 3.6 ms, acquisition matrix = 256 × 256, field of view (FOV) = 256 mm^2^, flip angle (FA) = 8°, slice thickness = 1.0 mm, and 185 sagittal slices.

### Data processing

The surface-based analysis was performed using the Computational anatomy toolbox (CAT12) (http://dbm.neuro.uni-jena.de/cat/), which is an automated approach based on a projection-based thickness (PBT) method to estimate the thickness of cortex and reconstruct the hemispheric central surface^28^. The procedures have been described in our previous study^29^. In brief, the processing pipeline included brain segmentation into gray matter, white matter and cerebrospinal fluid, affine registration to MNI template space, and subsequent nonlinear deformation. The cortical thickness was measured as follows: we used segmentation to estimate white matter distances and then project local maxima to other gray matter voxels using neighborhood relationships. We also extracted two additional measured surfaces, gyrification and sulcal depth. Based on the absolute mean curvature, gyrification index was calculated as a ratio of the external brain surface with the outer surface excluding the sulci^30^. The sulcus depth with square root function transformation, making the data more normally distributed, was defined as the Euclidean distance between the central surface and the convex hull. The square root function transformation was used to make the data more normally distributed. The newly generated images were smoothed using a Gaussian kernel with a full-width-half-maximum (FWHM) of 15 mm for cortical thickness and 20 mm for gyrification and sulcal depth, respectively.

### Statistical analyses

Two-sample t-tests were used to test the differences in age, education, and total intracranial volume, and a chi-square test was performed to assess the gender between the TN and HCs using SPSS 22.0.

The cortical morphological analyses were performed using two-sample t-tests on the left and right hemispheres separately, which included age, gender, and education as covariates. Multiple comparisons were conducted based on the family-wise error (FWE) method, with a cluster threshold of *p* = 0.001 and a corrected cluster significance of *p* < 0.05.

In addition, based on the results of the surface-based morphometry, we estimated the partial correlation between the mean cortical characteristics and the duration of TN, pain intensity, age of pain onset, while taking age, gender, and education as covariates (*p* < 0.05, uncorrected).

## Results

### Demographics and clinical characteristics

We found no significant differences between the TN and HC groups in age, gender, education and total intracranial volume (Table 1). The mean duration of pain in the patient group was 4.72 years, and the mean pain intensity was 8.83.

### Alterations of cortical morphology

#### Cortical thickness

**Figure 1:**
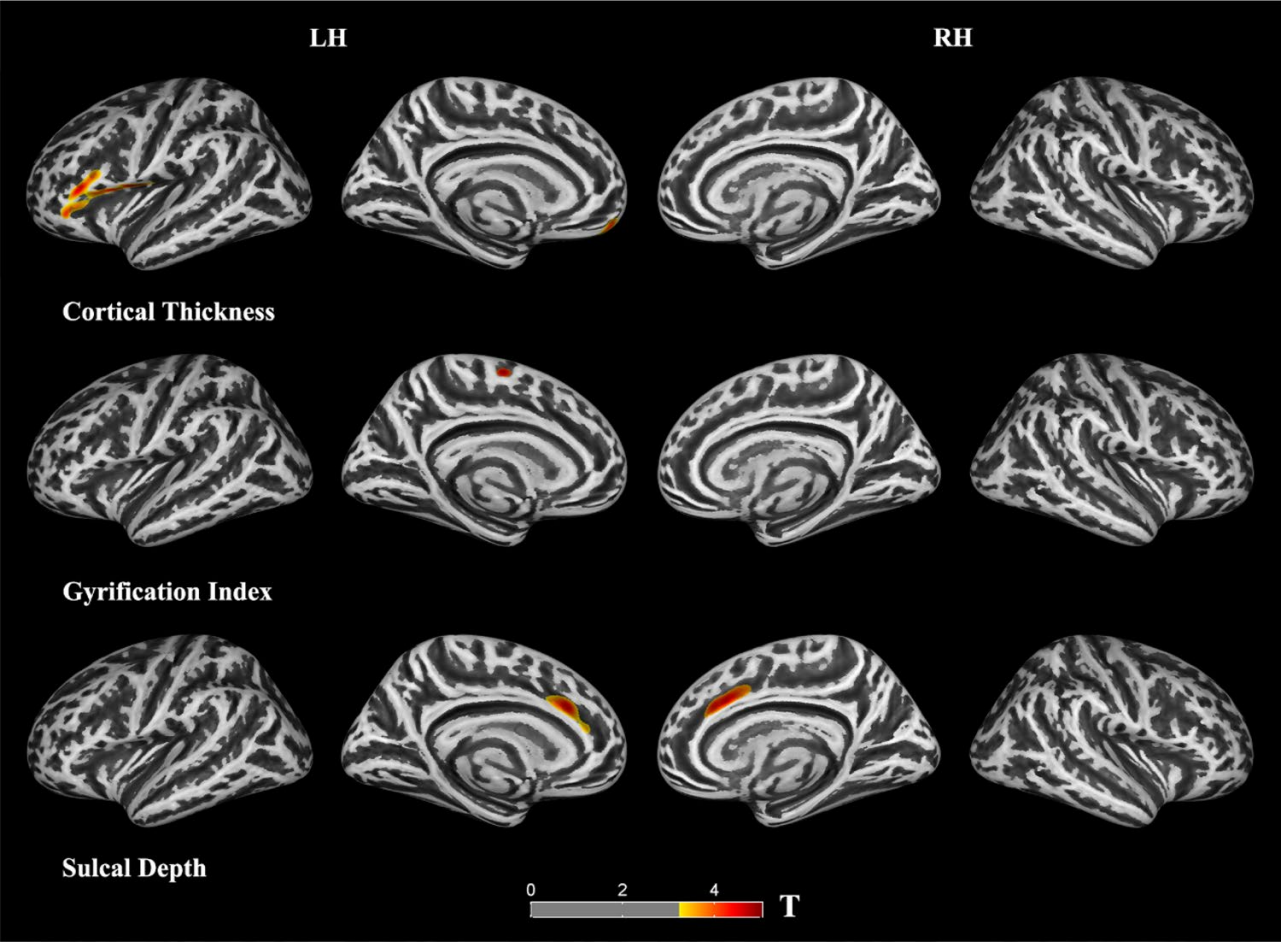
Clusters showing significantly changed cortical morphology in the trigeminal neuralgia (TN) patients compared to the healthy controls (HCs). LH (RH): left (right) hemisphere.

We found significant decreases of cortical thickness in left orbitofrontal and inferior frontal (extending to precentral) regions in the TN group compared to the HCs (*p* < 0.05, FWE corrected; Fig. 1, Table 2). Figure 1 shows the location of these clusters on the cortical surface, and Table 2 lists the detailed information.

**Table 2:** Clusters showing significantly changed cortical morphology in the trigeminal neuralgia (TN) patients compared to the healthy controls (HC).

Hemispheres/cortical morphology	Brain regions	Cluster size	T value	MNI		
				X	Y	Z
**Cortical thickness**						
LH	Inferior frontal	2509	4.6	− 53	30	8
	Precentral					
	Medial orbitofrontal	320	4.2	− 4	54	− 18
**Gyrification**						
LH	Superior frontal	127	5.3	− 8	− 11	27
**Sulcal depth**						
LH	Superior frontal	485	4.6	− 12	24	28
	Anterior cingulate					
RH	Superior frontal	838	4.3	11	17	34
	Anterior cingulate					

LH (RH), left (right) hemisphere.

#### Gyrification index

We found a significant decrease of gyrification in the left superior frontal gyrus in the TN patients (Fig. 1, Table 2).

#### Sulcal depth

We found significant decreases of sulcal depth in the bilateral superior frontal and anterior cingulate regions in the TN patients compared to the HCs.

### Quantification of alterations in cortical morphology

**Figure 2:**
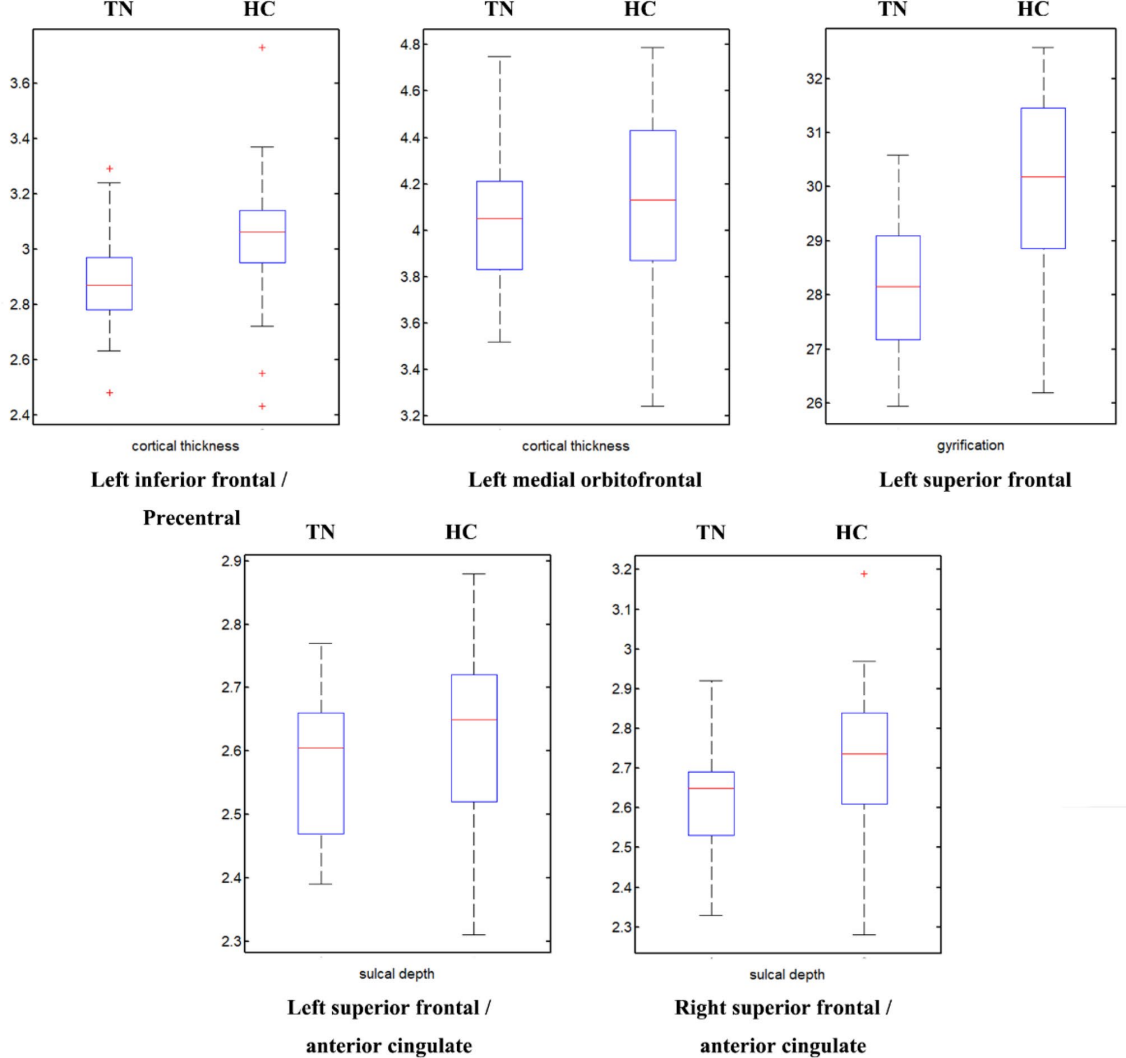
Box plots of clusters showing significantly changed cortical morphology in the trigeminal neuralgia (TN) patients compared to the healthy controls (HC).

For each cluster listed in Table 2, we also extracted the mean cortical characteristics for each individual, and then compared between these two groups. The comparison showed that cortical alteration in TN group ranged from 2.3 to 2.7% (Table 3 and Fig. 2).

**Table 3:** Quantification of clusters showing significantly changed cortical morphology in the trigeminal neuralgia (TN) patients compared to the healthy controls (HC).

Hemispheres/cortical morphology	Brain regions	Mean cortical characteristics		Difference in % (D)
		TN	HC	
**Cortical thickness**				
LH	Inferior frontal	2.88 ± 0.18	3.03 ± 0.24	5.0
	Precentral			
	Medial orbitofrontal	4.05 ± 0.31	4.15 ± 0.38	2.4
**Gyrification**				
LH	Superior frontal	28.17 ± 1.30	29.88 ± 1.73	5.7
**Sulcal depth**				
LH	Superior frontal	2.58 ± 0.11	2.64 ± 0.13	2.3
	Anterior cingulate			
RH	Superior frontal	2.63 ± 0.14	2.72 ± 0.18	3.3
	Anterior cingulate			

LH (RH), left (right) hemisphere. Difference in % (D) was calculated using the equation: D = (TN − HC)/HC.

### Correlation analysis

**Figure 3:**
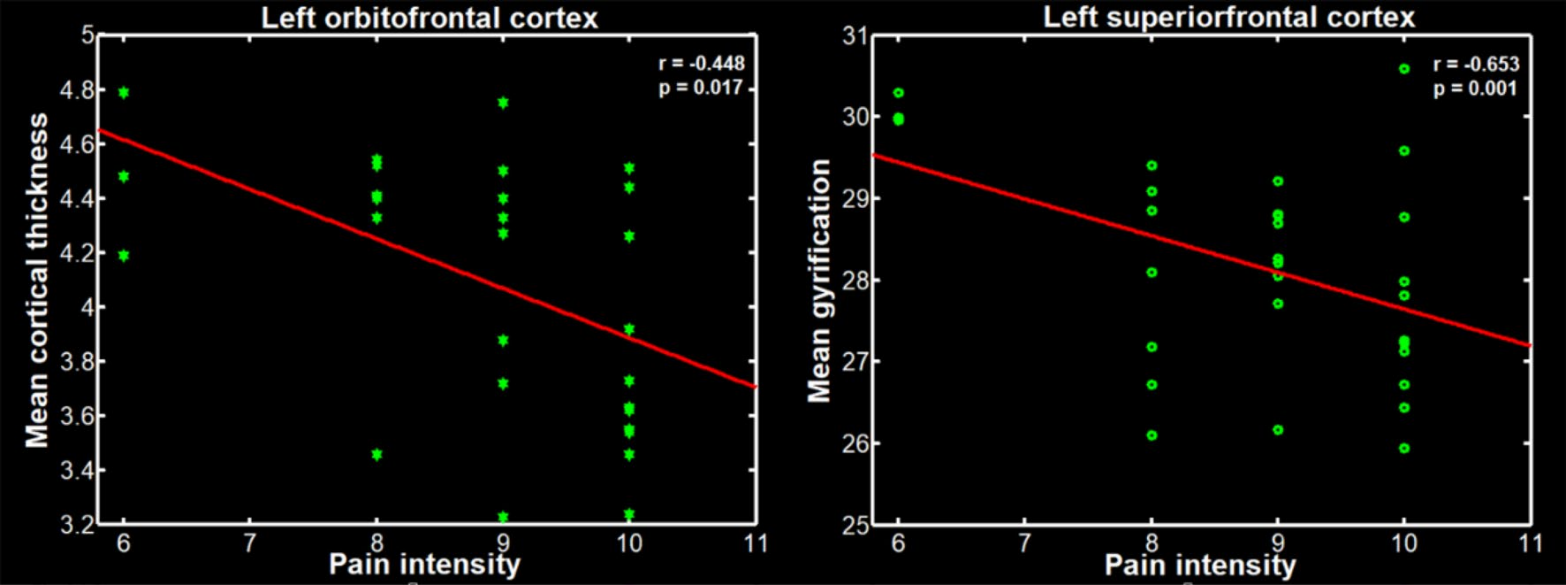
Scatter plots of the mean gyrification of the left superior frontal cortex, and the mean cortical thickness of the left orbitofrontal cortex negatively correlated with the pain intensity in the trigeminal neuralgia (TN) patients.

Correlation analysis found that in TN patients, a significant negative correlation was found between the mean cortical thickness of the left orbitofrontal cortex and pain intensity (*r* = − 0.448, *p* = 0.017, uncorrected). There was also a significant negative correlation between the mean gyrification index of the left prefrontal cortex and pain intensity (*r* = − 0.653; *p* = 0.001, uncorrected) (Fig. 3). However, there was no significant correlation between the mean values of significant cortical regions presented in Table 2 and the duration of disease.

## Discussion

In this study, a CAT12-based cortical morphometry was used to quantify changes in cortical thickness, gyrification index, and sulcal depth in the patients with trigeminal neuralgia. We found that the morphological changes of cortex were mainly located in the prefrontal cortex and anterior cingulate region (Fig. 1). In addition, we found negative correlations between mean cortical thickness/gyrification index and pain intensity.

We found that, compared to the control group, the cortical characteristics of TN patients, including decreased cortical thickness, gyrification index, and sulcal depth, were mainly located in the frontal region (Fig. 1 and Table 2). The superior frontal gyrus (extending to the anterior cingulate cortex), inferior frontal gyrus and orbitofrontal cortex belong to the prefrontal cortex, which plays an important role in emotion, cognitive processing and pain management^31–33^. FMRI studies have shown that mood and pain management abnormalities caused by chronic pain are associated with changes in prefrontal-cingulate neural network activation^5,34–36^. For example, Rottmann et al.^34^ evaluated the effect of low-frequency electrical stimulation and found brain activation in the insula, anterior cingulate cortex, superior temporal gyrus, and prefrontal cortex. A recent meta-analysis concluded that the major regions associated with structural and functional changes in TN include the frontal and cingulate cortex^5^. Brain morphological studies have also found abnormal structural changes in the frontal lobe and cingulate regions of pain patients^37–39^. For example, using VBM, Apkarian et al.^37^ found for the first time that gray matter density decreased in the bilateral prefrontal cortex in patients with chronic low back pain. Fritz et al.^38^ also reported decreased gray matter volume in the prefrontal cortex in chronic back pain patients, which was negatively correlated with pain intensity. And for TN patients, there are similar results^6,7,17,18^. Obermann et al.^7^ identified specific brain regions possibly related to TN and found decreased gray matter volume in prefrontal cortex, anterior cingulate gyrus, orbitofrontal cortex and other regions in TN patients compared with the healthy control group. Tsai et al.^6^ also reported that the volume of prefrontal cortex decreased in TN patients. Using surface-based morphometry, Parise et al.^17^ found that there were abnormal changes (uncorrected) in cortical thickness in the frontal region of TN patients. Desouza et al.^18^ found that the anterior cingulate cortex and orbitofrontal cortex were thinner in TN patients. A study of Schmidt-Wilcke et al.^39^ about chronic facial pain have shown a decrease in gray matter volume in the anterior cingulate region and demonstrated that the anterior cingulate connects the prefrontal cortex to the limbic system, which is associated with pain regulation. Thus, the observed decrease in cortical morphology in the prefrontal and anterior cingulate regions may reflect a highly pain-related response to chronic pain, including TN. Considering the functional correlation between the two regions, the interregional connections between the prefrontal cortex and the anterior cingulate cortex appear to form part of the central pain processing system, responsible for pain regulation and perception.

In addition, it was found that compared to the HC group, the cortical thickness decreased in the left precentral gyrus of TN patients. As a part of the primary motor cortex, the precentral gyrus is responsible for reflecting sensory pain response, maxillary motor inhibition, and facial muscle tension^40^. Hayes and Northoff^41^ previously reported that painful stimuli could activate the motor cortex. One previous study of Peck et al.^42^ has proposed and refined a new "Integrated Pain Adaptation Model", which stated that pain could induce a new recruitment strategy to change muscle activity to minimize pain. For TN patients, simple and painless exercise can also lead to trigeminal neuralgia attacks^43^. Therefore, TN patients would like to limit facial movement to reduce pain. Therefore, we hypothesized that inhibition of the pain response of the trigeminal nerve to relieve pain might result in plastic changes in the primary motor cortex.

The correlations between cortical morphology and pain intensity may indicate the effect of pain intensity on changes of the cerebral cortex. Our study found that there was a significant negative correlation between the mean cortical thickness of the left orbitofrontal cortex and pain intensity, and a significant negative correlation between the mean gyrification of the left superior frontal cortex and pain intensity. In other words, the higher the level of pain, the thinner the thickness of the cortex and the lower the gyrification index. This is similar to the results of previous study of chronic pain, which analyzed the changes in the volume of gray matter in the patients with back pain and found a negatively correlation between the decreased gray matter volume in the prefrontal cortex with pain intensity^38^. Therefore, the correlations between the cortical morphology of the frontal cortex and the intensity of pain may indicate that the intensity of pain is a key factor responsible for the morphological changes of TN patients.

In this study, we first reported the abnormal changes in the sulcal depth and gyrification. Previous studies have indicated that sulcal depth and gyrification reflects a measure of cortical folding and complexity^27^, which are sensitive to cortical development^22–24^. Thus, our finding of altered sulcal depth and gyrification may also provide the evidence of pain-related cortical morphological changes.

Unfortunately, we noticed lack of overlap in our findings when comparing the results among three cortical characteristics (Table 2). This apparent inconsistency in the locations of altered characteristics may have occurred due to the different procedures of the three morphologic indicators. In terms of calculation, cortical thickness was obtained by estimating the white matter distance and maximum local mapping. Gyrification values were based on absolute mean curvature. Square root-transformed sulcus depth was based on the Euclidean distance between the central surface and its convex hull^30^. In terms of spatial smoothing, cortical thickness was normalizing the cortical surface with a 15 mm Gaussian kernel, while sulcal depth and gyrification was used a 20 mm Gaussian kernel. The second possible interpretation is that three measures might be sensitive to different underlying neurobiological effects. Cortical thickness could reflect the structure of the cerebral column^10^. Sulcal depth might be related to genetic control and cytoarchitectonic areas^44^. Cortical gyrification allows a larger cortical surface area to fit in the skull, facilitating the development of compact neural circuits^27^. Generally speaking, the SBM analysis might provide a comprehensive perspective to the findings in TN.

Compared with other neuropathic pain, we noticed decreased cluster in the cingulate, insula and thalamus, frontal, temporal and postcentral gyrus^45–47^, which was partly consistent with our findings. For example, Mao et al.^46^ studied patients with chronic low back pain and found reported decreased gray matter volume in the bilateral superior frontal gyrus, right frontal pole, left insular cortex, left middle and inferior temporal gyrus. Blankstein et al.^47^ reported cortical thinning in the anterior midcingulate cortex in irritable bowel syndrome. The possible explanation for consistent findings is that different types of pain may have common pain mechanisms, as well as specific mechanisms associated with pain. However, as to the TN patients, limited studies on cortical morphology have not found consistent results^17,18,21^. For example, Parise et al.^17^ found reduced cortical thickness in the left cuneus and left fusiform in the TN patients. In contrast, the study of DeSouza et al.^18^ showed both cortical thickening and thinning compared to controls. Wang et al.^21^, reported significant reductions of local gyrification index in the left insular cortex. And our study found that TN patients exhibited significantly reduced cortical thickness in the left inferior frontal, and left medial orbitofrontal cortex; reduced gyrification in the left superior frontal cortex; and reduced sulcal depth in the bilateral superior frontal cortex. The reason for these inconsistent results in the cortical morphometry may be multifaceted, such as the precise patient samples studied or the methods used, and need to be further investigated.

The study has some limitations. Firstly, we adopted a cross-sectional design, and detected reductions in cortical morphological indicators in patients with TN. However, we cannot elucidate the causal relationship between alterations of cortical characteristics and TN development. Secondly, all TN patients in this study were treated with analgesics, therefore, we cannot rule out the possible confounding effect of drugs in cortical morphological analysis. Thirdly, the inhomogeneous distribution of affected sides (8 left/22 right) in the included subjects may be responsible for the left-lateralization of cortical morphometry, which may indicate possible compensatory effects or neuroadaptation. Fourthly, in the correlation analyses, the uncorrected results were presented, which may provide the possible correlation between cortical characteristics and pain intensity in TN. Future studies should expand the sample size and report the corrected results to increase the statistical efficiency of correlation analysis.

## Conclusions

In conclusion, this is the first comprehensive analysis of cortical thickness, gyrification index, and sulcal depth in patients with trigeminal neuralgia. We found that patients with trigeminal neuralgia had cortical morphological changes in prefrontal cortex, anterior cingulate and precentral gyrus, which are mainly involved in pain management and perception. This study also found a correlation between reduced cortical morphological indicators and increased pain intensity. These results may provide important information about the neurophysiological mechanisms associated with pain.

## Data Availability

All data generated or analysed during this study are included in this published article.
